# Arterial, Venous, and Cerebrospinal Fluid Flow and Pulsatility in Stroke-Related Cerebral Small Vessel Disease: A Longitudinal Analysis

**DOI:** 10.1161/STROKEAHA.124.049103

**Published:** 2025-06-18

**Authors:** Alasdair G. Morgan, Michael J. Thrippleton, Michael S. Stringer, Francesca M. Chappell, Maria C. Valdés-Hernández, Stewart Wiseman, Lucia Ballerini, Rosalind Brown, Yajun Cheng, Xiaodi Liu, Junfang Zhang, Eleni Sakka, Daniela Jamie Garcia, Emilie Sleight, Cameron Manning, Roberto D. Coello, Dominic Job, Angela Jochems, Carmen Arteaga Reyes, Una Clancy, Ian Marshall, Fergus N. Doubal, Joanna M. Wardlaw

**Affiliations:** Department of Neuroimaging Sciences, Centre for Clinical Brain Sciences, The University of Edinburgh, United Kingdom (A.G.M., M.J.T., M.S.S., F.M.C., M.C.V.-H., S.W., R.B., E. Sakka, D.J.G., E. Sleight, C.M., R.D.C., D.J., A.J., C.A.R., U.C., I.M., F.N.D., J.M.W.).; UK Dementia Research Institute at The University of Edinburgh, Edinburgh Medical School, United Kingdom (A.G.M., M.J.T., M.S.S., F.M.C., M.C.V.-H., S.W., R.B., E. Sakka, D.J.G., E. Sleight, C.M., R.D.C., D.J., A.J., C.A.R., U.C., I.M., F.N.D., J.M.W.).; University for Foreigners of Perugia, Italy (L.B.).; Department of Neurology, West China Hospital of Sichuan University, Chengdu (Y.C.).; Division of Neurology, Department of Medicine, The University of Hong Kong, China (X.L.).; Department of Neurology, Shanghai General Hospital, Shanghai Jiao Tong University School of Medicine, China (J.Z.).

**Keywords:** carotid artery, internal, cerebral arteries, cerebral small vessel diseases, cranial sinuses, extracellular fluid, stroke, lacunar, vascular stiffness

## Abstract

**BACKGROUND:**

Cerebral small vessel disease (SVD) causes up to 45% of dementias and 25% of ischemic strokes, but the understanding of vascular pathophysiology is limited. We aimed to investigate the contribution of pulsatility of intracranial arteries, veins, and cerebrospinal fluid (CSF) and cerebral blood flow to long-term imaging and clinical outcomes in SVD.

**METHODS:**

We prospectively recruited participants in Edinburgh/Lothian, Scotland, with lacunar or nonlacunar ischemic stroke (modified Rankin Scale score ≤2, as controls) and assessed medical and brain magnetic resonance imaging characteristics at baseline and 1 year (2018–2022). We used phase-contrast magnetic resonance imaging to measure flow and pulsatility in major cerebral vessels and CSF to investigate independent associations with baseline white matter hyperintensity (WMH) and perivascular space (PVS) volumes and their progression, as well as with recurrent stroke, functional, and cognitive outcomes at 1 year. We applied linear, logistic, and ordinal regression models in our analysis.

**RESULTS:**

We recruited 210 participants; 205 (66.8% male; aged 66.4±11.1 years) had useable data. In covariate-adjusted analyses, higher baseline arterial pulsatility was associated with larger volumes of baseline WMH (B=0.26 [95% CI, 0.08–0.44]; *P*=0.01) and basal ganglia PVS (B=0.12 [95% CI, 0.04–0.20]; *P*<0.01) but not with their change at 1 year (WMH: B=0.01 [95% CI, −0.05 to 0.06]; *P*=0.78; basal ganglia PVS: B=0.02 [95% CI, −0.04 to −0.07]; *P*=0.62) or cognition, dependency, or recurrent stroke at 1 year. Neither cerebral blood flow nor CSF pulsatility was related to baseline SVD severity, WMH/PVS progression, or clinical outcomes at 1 year.

**CONCLUSIONS:**

Associations between vascular/CSF pulsatility, cerebral blood flow, WMH/PVS, and clinical SVD features are complex. The lack of association between intracranial arterial, venous, or CSF pulsatility, cerebral blood flow, and WMH or PVS longitudinal change in this large, covariate-adjusted analysis questions the presumption that high intracranial vascular pulsatility causes SVD and its progression, consistent with other recent longitudinal studies. Intracranial pulsatility may differ from systemic vascular measures in their cause-pathogenic role(s) in SVD and should be considered separately.

Arterial stiffening is associated with several vascular pathologies, including stroke^1^ and cerebral small vessel disease (SVD).^2^ Arterial stiffness is increased by elastin loss, collagen replacement, and other factors,^3^ reducing vessel compliance.^4^ The resulting higher pulsatile stress can be measured in vivo using phase-contrast magnetic resonance imaging (PC-MRI) in larger blood vessels and may be transmitted to downstream blood vessels, causing tissue damage.^5^

Increased intracranial vessel pulsatility is associated with SVD.^5–8^ However, some studies show that neither cerebral blood flow (CBF)^9^ nor middle cerebral artery,^10^ internal carotid arteries (ICAs),^11^ or small distal cerebral artery pulsatility^11^ predicts white matter hyperintensity (WMH) progression. An increase in systemic large artery stiffness, measured via carotid-femoral pulse wave velocity (PWV), was the only vascular factor that occurred before the progression of small vessel disease (SVD) or cognitive decline.^12^

Cerebrospinal fluid (CSF) dynamics are integral to intracranial fluid physiology and can be assessed contemporaneously by PC-MRI, providing more information about brain compliance and fluid homeostasis.^13^ Animal models suggest that vessel pulsatility may modulate CSF flow, mixing with interstitial fluid, and facilitate brain interstitial fluid and metabolic waste clearance via meningeal lymphatics.^14^

We aimed to examine cranial arterial,^6^ venous,^8^ and CSF^15–17^ flow and pulsatility and clarify their cross-sectional and longitudinal associations with SVD burden and clinical outcomes. We examined how measures of intracranial vessel stiffness were associated with SVD severity on imaging at baseline and 1-year clinical outcomes. We aimed to assess (1) how MRI-derived cerebrovascular stiffness associates with concurrent SVD features and (2) if altered baseline blood/CSF flow and pulsatility predict more severe 1-year SVD burden, cognitive decline, disability, or recurrent stroke or transient ischemic attack or incident infarcts on MRI.

## Methods

The prospective MSS3 (Mild Stroke Study 3; International Standard Randomised Control Trial Number [ISRCTN]:12113543) studies cross-sectional and longitudinal mechanisms, imaging, and clinical features of sporadic SVD.^18^ Full details of participant recruitment, assessments, and follow-up are published.^18–20^ Data underpinning this study and for the MSS3 in general are available upon written request to the corresponding author. In brief, we recruited patients with mild ischemic stroke (ie, nondisabling and modified Rankin Scale (mRS) score ≤2) of lacunar or nonlacunar (ie, minor cortical) ischemic stroke subtypes from National Health Service (NHS) Lothian, Scotland stroke services from 2018 to 2021 and followed them through 1 year (to 2022). Study recruitment and baseline assessment took place between August 2018 and October 2021, while completion of 1-year follow-up was in October 2022. The South East Scotland Research Ethics Committee (reference 18/SS/0044) approved the study, and all participants provided written informed consent. All patients received UK guideline stroke prevention therapies including antiplatelet, lipid-lowering, and antihypertensive drugs as appropriate. Those with poorly controlled heart rates were excluded from the study. Data acquisition and image analysis were blind to pulsatility/CBF measures, and all methods were validated.^18,21–27^ Per *Stroke* guidelines, we report against STROBE (Strengthening the Reporting of Observational Studies in Epidemiology).

We included the nonlacunar ischemic strokes as a comparator group for the lacunar strokes because both lacunar and nonlacunar strokes all receive the same secondary prevention drugs and have similar vascular risk factors of hypertension, smoking, hyperlipidemia, and diabetes, and we wished to control for the effect that these drugs or common risk factors might have on the pulsatility (and other vascular function) measures. All strokes were assessed for severity of SVD (WMH, lacunes, perivascular space [PVS], microbleeds, atrophy, and summary SVD score).

At baseline, we recorded hypertension, diabetes, hypercholesterolemia diagnosis, and smoking history. We assessed dependency (mRS)^28^ and cognition (Montreal Cognitive Assessment)^29^ at baseline and follow-up. We recorded any recurrent ischemic event (ie, new stroke/transient ischemic attack or MRI incident infarct) between baseline and follow-up.

We recorded supine blood pressure (BP) before baseline MRI and calculated pulse pressure as systolic BP minus diastolic BP. We did not gather data on BP variation in this cohort as it was beyond the scope of this study.

### Magnetic Resonance Imaging

All participants underwent diagnostic imaging at the initial stroke presentation. At 1 to 3 months poststroke (to avoid acute effects) and 1 year, we scanned participants using 3T MRI (MAGNETOM Prisma, Siemens Healthcare, Germany) with 3D T1w, T2w, fluid-attenuated inversion recovery, susceptibility-weighted imaging, and diffusion-weighted imaging (for full details, see Supplemental Methods and protocol article).^18^

**Figure 1.**
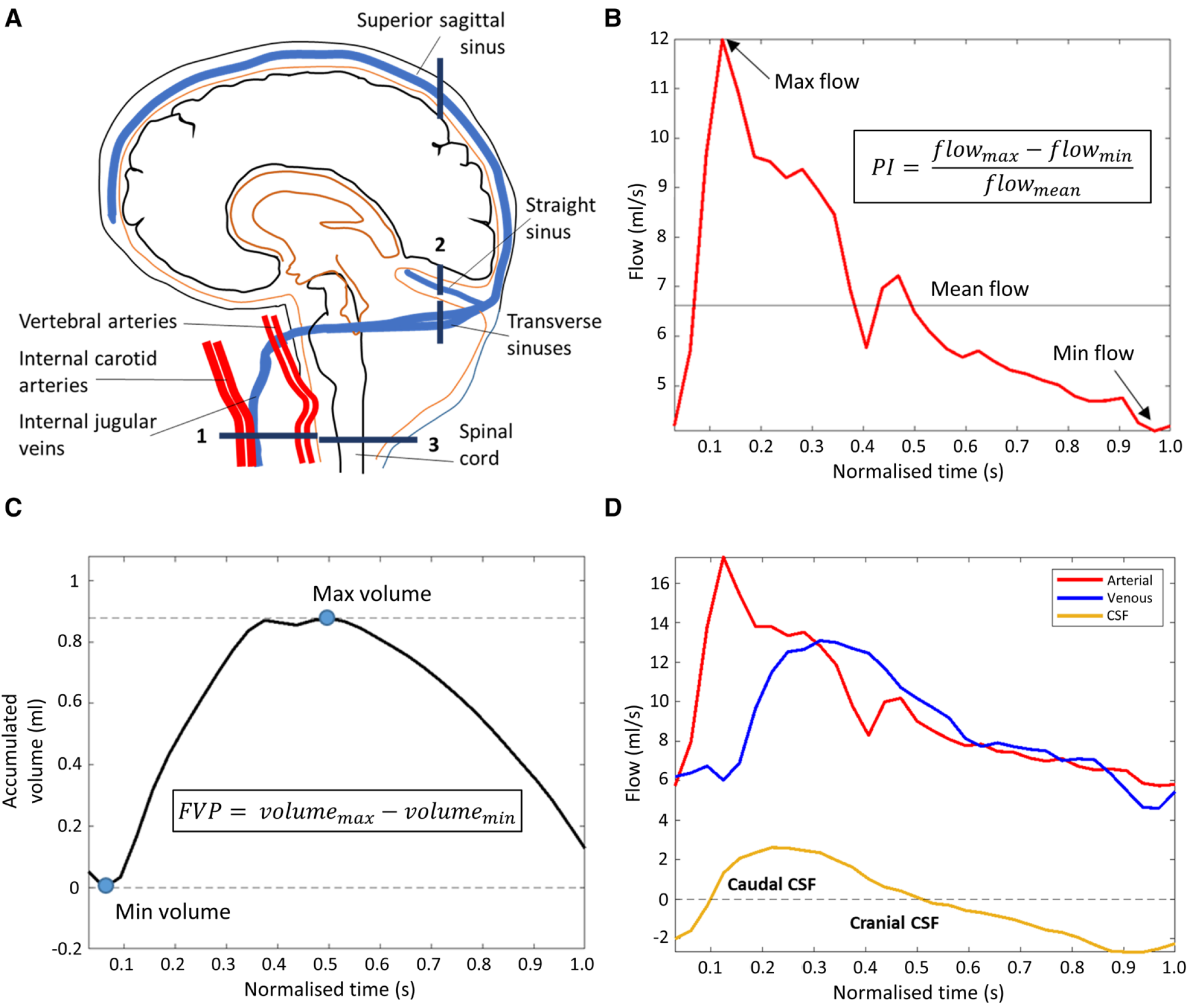
**Measuring intracranial flow. A**, Phase-contrast magnetic resonance imaging slice locations: 1, an axial slice across the jugular veins, carotid, and vertebral arteries at C2-3 level of the spine; 2, a coronal slice across the venous sinuses; and 3, an axial slice across the spinal cord at C2-3 level. **B**, Internal carotid artery blood flow (mL/s) across the cardiac cycle. The horizontal line indicates the mean flow. **C**, The cumulative integrated flow volume across the cardiac cycle, after subtraction of the mean flow rate from the flow waveform and shifted to set minimum volume to zero. **D**, Absolute arterial, venous, and cerebrospinal fluid (CSF) flow across the cardiac cycle. Arterial inflow is followed by a rise in venous outflow, and more rapid efflux of CSF, to maintain pressure equilibrium. As blood drains via the veins, CSF flow reverses. FVP indicates flow volume pulsatility.

At baseline only, we performed 3 separate 2D PC-MRI acquisitions to assess carotid arteries, venous sinuses, and CSF spaces (Figure 1A): one axial slice perpendicular to the external carotid arteries and ICAs at the spine C2-3 level (repetition time/echo time [TR/TE]=19.6/5.8 ms; flip angle, 12°; spatial resolution, 1.0×1.0 mm^2^; temporal resolution, 39.2 ms; and v_enc_=70 cm/s), one coronal slice bisecting the superior sagittal sinus, straight sinus, and transverse sinuses (TR/TE=21.7/6.6 ms; flip angle, 12°; spatial resolution, 0.71×0.71 mm^2^; temporal resolution, 43.4 ms; and v_enc_=50 cm/s), and one axial slice perpendicular to the C2-3 level spinal cord (TR/TE=25.2/8.5 ms; flip angle, 12°; spatial resolution, 0.83×0.83 mm^2^; temporal resolution, 50.4 ms; and v_enc_=6 cm/s). We interpolated the phase images across 32 timeframes, covering the cardiac cycle, using retrospective cardiac gating, a well-established multicenter study and trial method.^8,30,31^

### Image Analysis

#### Arterial, Venous, and CSF Flow Rates

We calculated the maximum PC-MRI signal magnitude across the cardiac cycle per pixel to help with vessel identification and drew regions of interest (ROIs) for the ICAs, vertebral arteries, internal jugular veins, superior sagittal sinus, straight sinus, and transverse sinuses lumens and subarachnoid CSF space at the foramen magnum using established methods.^8,18^ For each vessel, we calculated pixel velocities per timeframe using in-house code (Matlab, version 9; MathWorks, Inc, United States), correcting for background error by subtracting the mean velocity calculated across an ROI placed over nearby stationary tissue.^32^ We multiplied velocities by pixel area and summed them to calculate the blood flow rate in mL/s to obtain a flow waveform across the cardiac cycle (Figure 1B).

We calculated CBF (mL/min) as the mean inflow (ICAs and vertebral arteries) per minute, normalized to brain volume^18^ to provide CBF/min per 100-mL brain volume.^8,18^ We calculated the pulsatility index (PI) and the resistance index (RI) using modified Gosling^33^ and Pourcelot^34^ equations (PI=flow_max_−flow_min_/flow_mean_; RI=flow_max_−flow_min_/flow_max_; Figure 1B). We calculated combined arterial PI by summing flow waveforms across vertebral arteries and ICAs before using the equation. We calculated combined venous sinus PI as the mean of the PI values from each relevant vessel (summing waveforms was not appropriate due to them either being downstream of each other or originating from separate areas of the brain; Figure 1A). This was done for RI and flow volume pulsatility (FVP) also.

We calculated pulse transit time (PTT; a PWV indicator)^8^ by normalizing the cardiac cycle to 1 second and calculating the time difference between peak ICA and downstream vessel flow.^8,35^ For subarachnoid CSF PTT, only peak caudal flow was considered, as it corresponds to arterial inflow.^36^ We excluded PTT measurements in cases where there was a difference in patient heart rate of >15 bpm between the relevant scans.

For blood flow volume analysis in all arteries and veins ROIs, we subtracted mean flow and plotted cumulative integration (Figure 1C). We calculated FVP (unitless) by subtracting minimum from maximum volume change across the cardiac cycle (FVP=volume_max_–volume_min_).^5^ We calculated net CSF flow by integrating the cranial (positive) and caudal (negative) flow values across the cardiac cycle, expressing net flow in mL/min,^30^ and calculated peak CSF flow (mL/s) as the absolute maximum flow rate. We also calculated CSF stroke volume (mL), that is, average absolute flow volume.

#### Quantitative SVD Lesion Assessment

We used well-validated computational methods to measure baseline intracranial volume (ICV) and baseline and 1-year brain, WMH, and PVS volumes.^21–23^ In brief, using FSL-FLIRT, we coregistered all scans to the T2-w image. We determined ICV computationally by brain extraction from the susceptibility-weighted image. To determine WMH volume, we used an established pipeline^18,22,23^ to apply intensity-based thresholding to the fluid-attenuated inversion recovery scan and excluded false positives around the choroid plexus, aqueduct, third and fourth ventricles, using Freesurfer (https://surfer.nmr.mgh.harvard.edu/). In addition, to exclude hyperintensities unlikely to reflect pathology, we applied a lesion distribution probabilistic template to the thresholded images.^23^ We manually excluded index and old infarcts and normalized WMH volume as %ICV. PVS volumes (mL) were segmented from the T2-w image using an established filter-based approach^23^ in the basal ganglia (BG) and centrum semiovale (CSO) ROIs,^21,23^ and reported as % ROI volume. All masks were checked by experienced image analysts (S.W. and M.C.V.-H.) specialized in SVD and stroke and checked by J.M.W. Viewing was performed in high-quality neuroradiological standard viewing software reviewing the diffusion, fluid-attenuated inversion recovery, T2, and susceptibility-weighted imaging to ensure accuracy of manual delineation of the acute and any old nonlacunar (cortical) or small subcortical infarcts. We visually rated structural images using the STRIVE-1 criteria (Standards for Reporting Vascular Changes on Neuroimaging 1 ).^25^ We assessed periventricular and deep WMHs (Fazekas scale),^26^ BG and CSO PVS score,^25^ and the presence of microbleeds and lacunes to compute summary SVD score (0–4).^27^ Scores were rated by 4 raters (X.L., J.Z., Y.C., and D.J.G.), and all readings were checked by an experienced neuroradiologist (J.M.W.).

### Statistical Analysis

We performed all statistical analyses using R (version 3.6.1, Austria) in RStudio (version 1.2.5019, RStudio, Inc, United States). We assessed model diagnostics (histograms, QQ and heteroscedasticity plots, residual versus fitted values, and multicollinearity) to verify relevant assumptions. To give normally distributed residuals, we log10 transformed WMH (% ICV) and PVS (% ROI).

To assess factors associated with pulsatility, we used multivariable linear regression models, each with a flow/stiffness measure as the outcome and clinical features (ie, hypertension, diabetes, hypercholesterolemia diagnosis, and smoking history [ever/never] as predictors). To assess the relation of pulsatility measures to continuous imaging outcomes, we used flow/stiffness measures as predictor variables and SVD-related measures as outcome variables. All linear models are reported as regression coefficient of interest (B), 95% CI, and *P* value.

For binary (eg, recurrent stroke/transient ischemic attack) and ordinal (eg, SVD score) outcomes, we used logistic regression models and reported odds ratios (ORs) and 95% CIs. While *P* values are reported for ordinal logistic models, note that their interpretation can be challenging.^37^ We grouped mRS categories with sparse data (scores, 2–5) to ensure there were sufficient data per category and satisfy the proportional odds assumption. As only 2 patients had died (ie, mRS score, 6) and composite outcomes including death may be problematic,^38^ we excluded them from mRS analyses.

To assess longitudinal relationships, we used the follow-up variable of interest as the outcome, with the baseline value as an additional covariate.

Models were adjusted for age, sex, systolic BP, and baseline WMH volume/ICV %, consistent with previous work.^30^ For baseline cross-sectional pulsatility analysis, we used all available arterial and venous stiffness measures (PI, RI, PTT, and FVP) in separate regression models but only PI in longitudinal pulsatility analyses, following a similar study.^11^

We did not correct for multiple comparisons in this exploratory study but instead cautiously interpreted significance levels.

## Results

### Population

**Figure 2.**
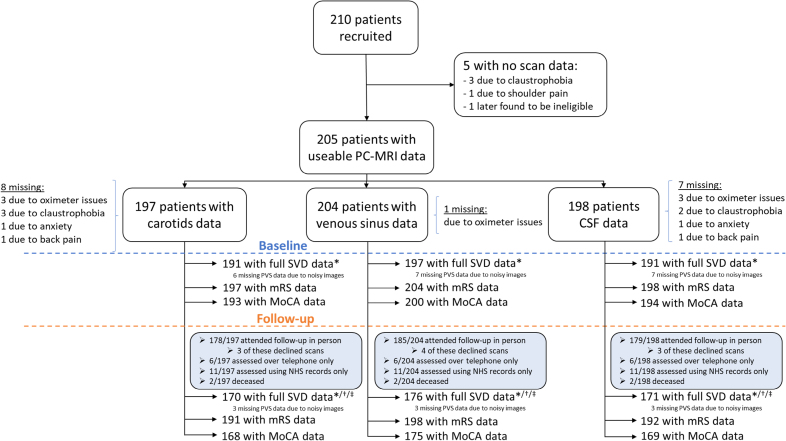
**Patient flowchart for useable data.** Of the 210 patients recruited, 205 had partially complete flow data due to the reasons listed. We have shown the numbers of patients with data at baseline and follow-up visits. *Computational quantitative measurements, including brain volume. †One patient missing perivascular space (PVS) measurements due to a lack of a T2w scan. ‡One patient missed the microbleed assessment, and, therefore, SVD score, due to lack of susceptibility weighted imaging (SWI) scan. CSF indicates cerebrospinal fluid; MoCA, Montreal Cognitive Assessment; MRI, magnetic resonance imaging; mRS, modified Rankin Scale; NHS, National Health Service; PC, phase contrast; and SVD, small vessel disease.

We recruited 210 patients, of whom 205 (66.8% male; mean age, 66.4±11.1 years; Table 1) had usable baseline PC-MRI flow data (Figure 2). Ten of 205 patients had partial flow data and 198 full baseline SVD imagings. By 1 year, 2 patients had died, 186 of 203 attended follow-up assessment (mean, 380±37 days after baseline; 4 declined scans), and 17 were assessed via telephone/health records. For each analysis, we used all available data.

**Table 1.**
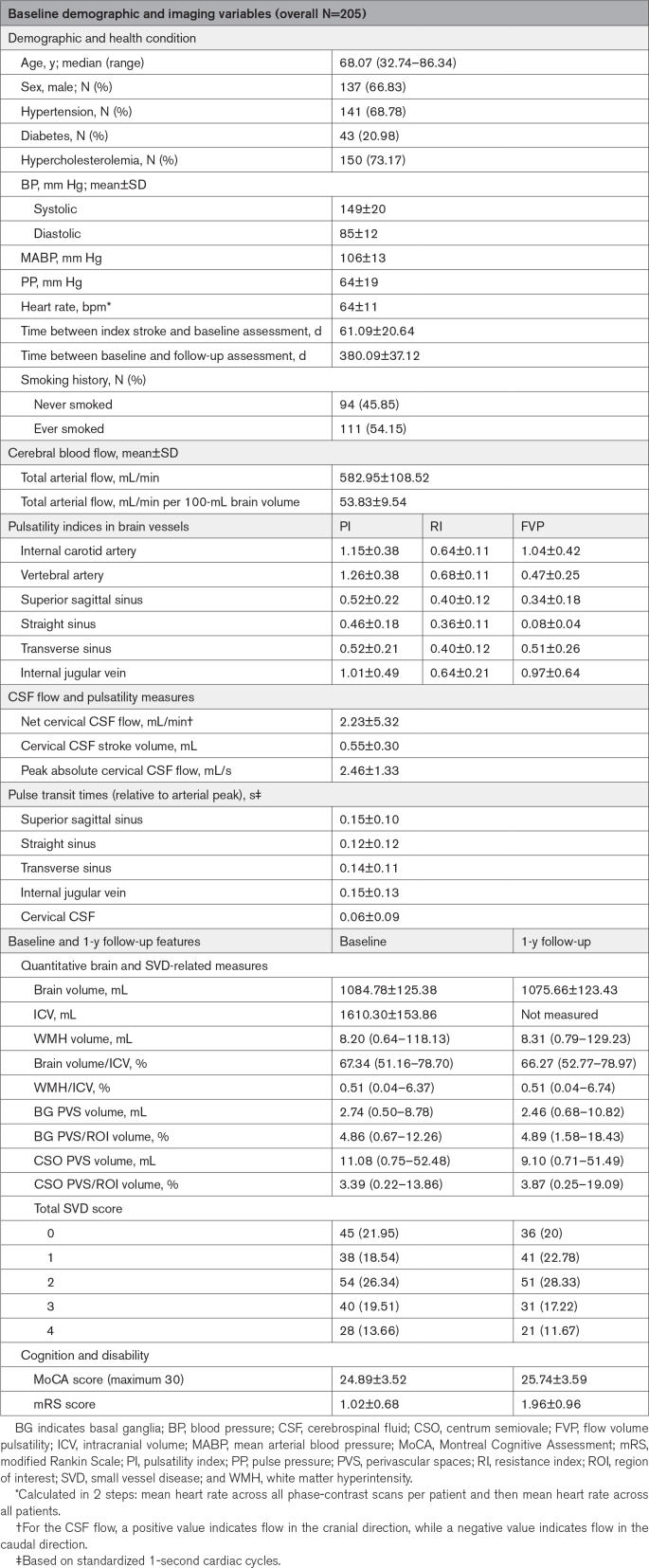
Patient Summary Statistics

Due to heart rate differences of >15 bpm between pairs of PC-MRI acquisitions, we excluded 7 patients from arterial-venous sinus PTT analysis and 7 patients from arterial-CSF PTT analysis.

We report baseline demographics, PC-MRI, baseline and 1-year quantitative brain volumes, SVD burden, and cognitive and disability measures summary statistics in Table 1. Although not used in the analyses here, Fazekas and PVS scores are shown in Table S1.

### Baseline Cross-Sectional Associations

In separate models, we found that patients with higher arterial PI tended to have more WMH (B=+0.260 [95% CI, 0.077–0.442]), BG PVS (B=+0.116 [95% CI, 0.038–0.195]), and CSO PVS volume (B=+0.094 [95% CI, −0.017 to 0.206]; Table 2). Patients with higher arterial PI tended to be older and have higher pulse pressure, hypertension, and diabetes (Tables 3 and 4).

**Table 2.**
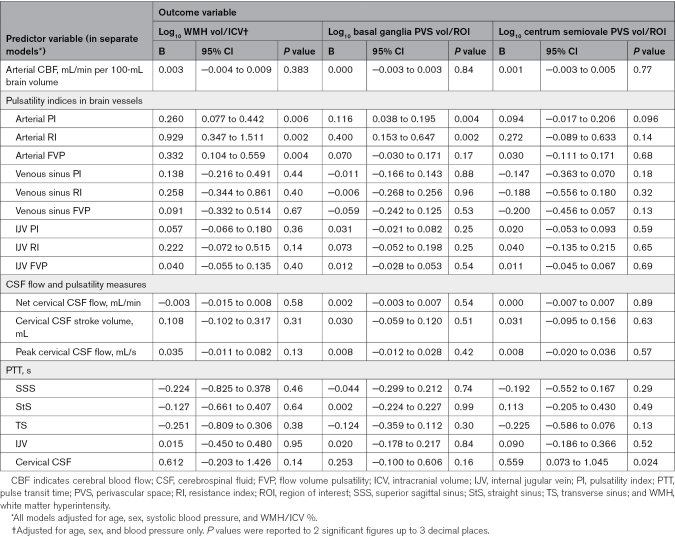
Regression Coefficients From Multivariable Baseline Analysis: Features of Cerebral Small Vessel Disease

**Table 3.**
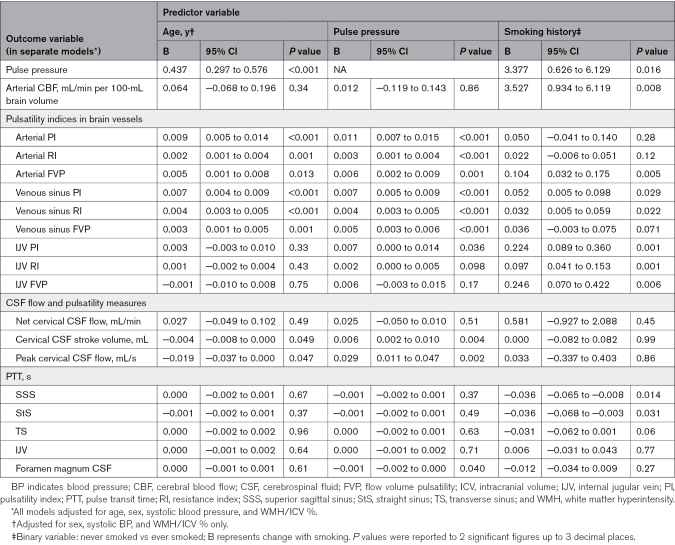
Regression Coefficients From Multivariable Baseline Analysis: Age, Pulse Pressure, and Smoking History

**Table 4.**
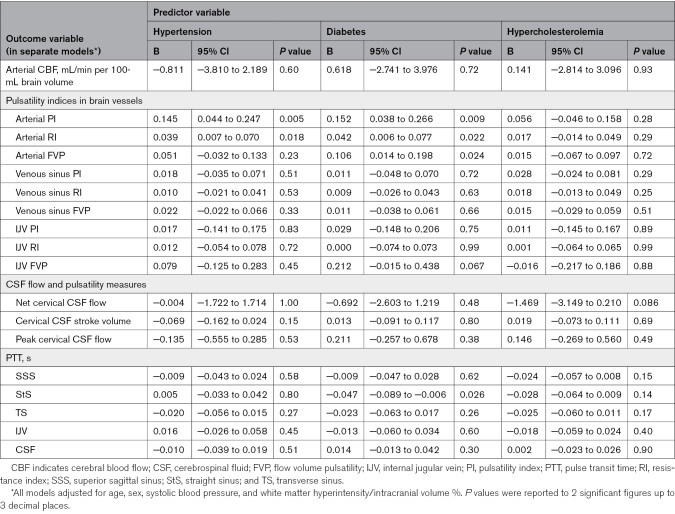
Regression Coefficients From Multivariable Baseline Analysis: Hypertension, Diabetes, and Hypercholesterolemia

Mean venous sinus PI differed little with WMH, BG PVS, or CSO PVS volumes (eg, WMH: B=+0.138 [95% CI, −0.216 to 0.491]). Patients with higher mean venous sinus PI tended to be older, have higher pulse pressure, and be current/ex-smokers.

Patients with longer PTTs between the arteries and cervical CSF exhibited larger CSO PVS volumes (B=+0.559 [95% CI, 0.073–1.045]; Table 2), while current/ex-smokers had shorter PTTs in the venous sinuses.

Where we found associations with PI, we generally found similar associations with RI (eg, WMH≈arterial RI: B=+0.929 [95% CI, 0.347–1.511]; Table 2), while higher FVPs associated with larger WMH volume (eg, WMH≈arterial FVP: B=+0.332 [95% CI, 0.104–0.559]) associations with PVS volumes were uncertain (eg, BG PVS≈arterial FVP: B=+0.070 [95% CI, −0.030 to 0.171]).

We found limited evidence of associations between CBF and any structural SVD-related measure (eg, WMH≈CBF: B=+0.003 [95% CI, −0.004 to 0.009]; Table 2), but current/ex-smokers had higher CBF (Table 3).

We found little association between CSF flow or pulsatility measures and structural SVD features (Table 2).

### Longitudinal Outcomes

**Figure 3.**
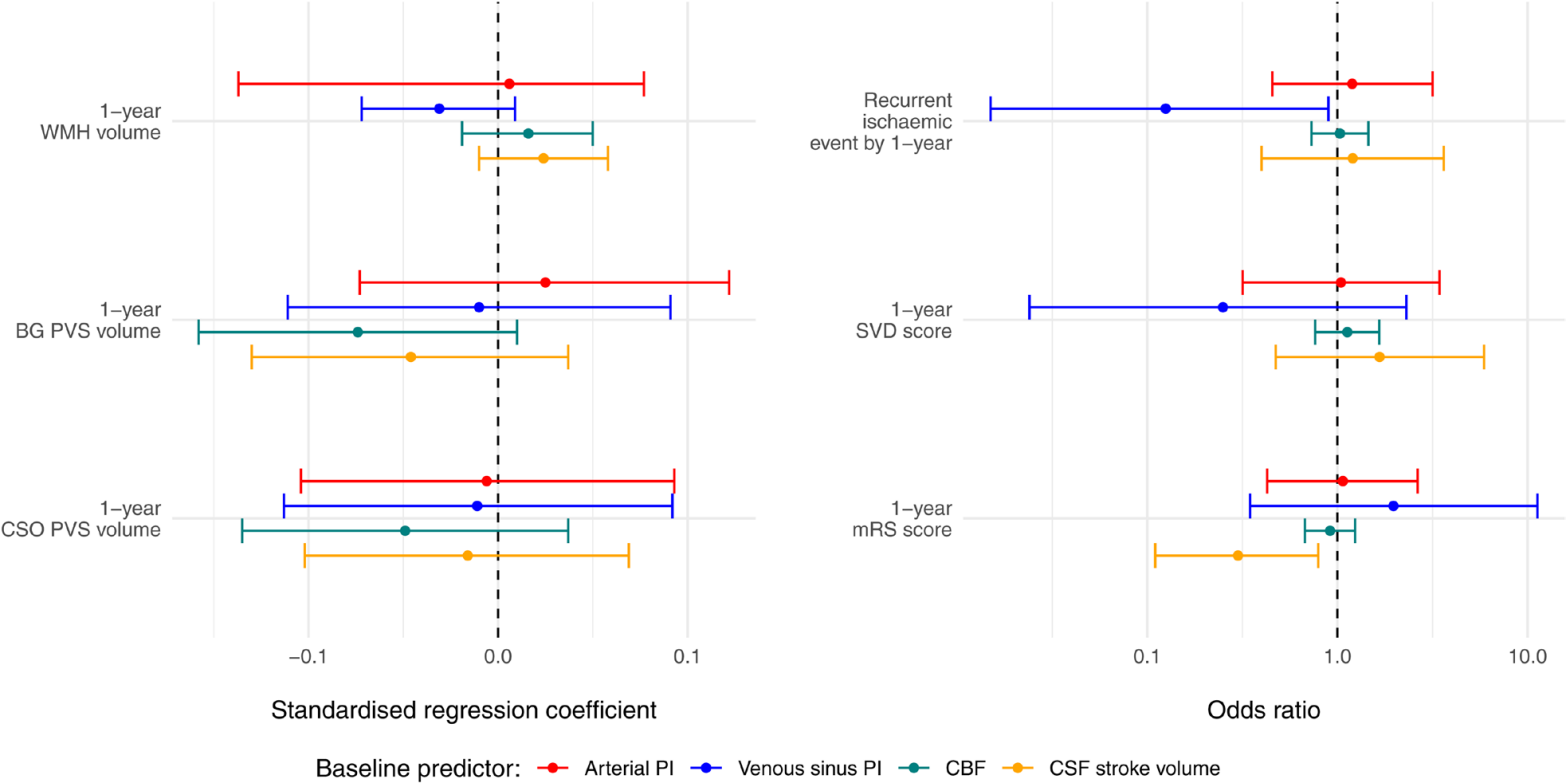
**Forest plot summarizing longitudinal flow/pulsatility index (PI) and small vessel disease (SVD) feature analyses. A**, Standardized regression coefficients between SVD features and arterial PI, mean venous sinus PI, cerebral blood flow (CBF), and cerebrospinal fluid (CSF) stroke volume. The dots represent the standardized coefficients, and the solid lines represent 95% CIs. **B**, Odds ratios on a log10 scale between intracranial flow and pulsatility measures and SVD, dependency, and recurrent ischemic event ordinal and binary outcomes. Dots show odds ratios, and solid lines show 95% CIs. Note that for visualization, we scaled the odds ratio of CBF in tens of mL/min per 100-mL brain volume. BG indicates basal ganglia; CSO, centrum semiovale; mRS, modified Rankin Scale; PVS, perivascular space; and WMH, white matter hyperintensity.

We did not find definite associations between 1-year SVD burden and baseline arterial PI, venous sinus PI, total CBF, or CSF measures (Table 5; Figure 3), for example, 1-year WMH≈arterial pulsatility: B=+0.008 (95% CI, −0.048 to 0.064). However, we found weak associations between higher 1-year BG PVS volume and both lower CBF (B=−0.002 [95% CI, −0.004 to 0.000]) and higher net foramen magnum CSF flow (B=+0.002 [95% CI, −0.001 to 0.006]). Patients with higher 1-year WMH volume tended to have higher CSF peak flow (B=+0.012 [95% CI, −0.001 to 0.025]).

**Table 5.**
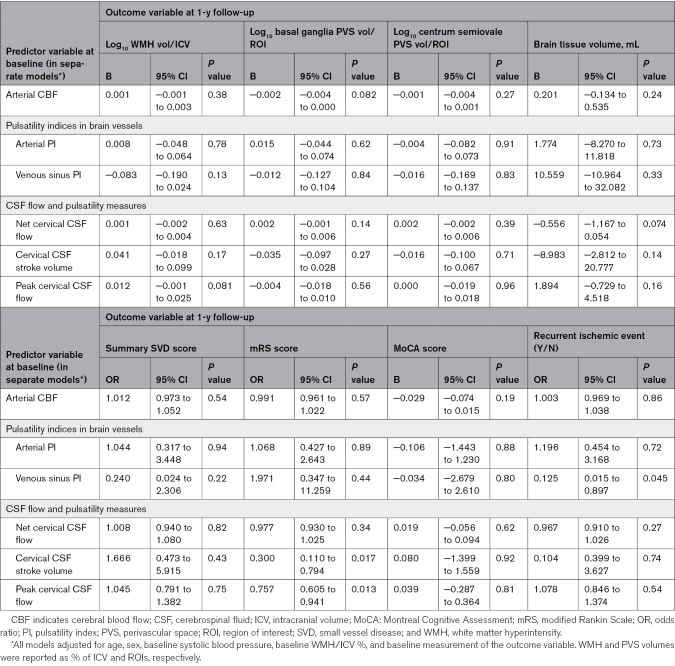
Adjusted Regression Analyses for SVD-Related Volumes at 1 Year

We found limited associations between baseline vascular flow/pulsatility measures and 1-year clinical outcomes (eg, 1-year mRS≈arterial PI: OR, 1.068 [95% CI, 0.472–2.643]), cognition (eg, 1-year Montreal Cognitive Assessment≈arterial PI: B=−0.106 [95% CI, −1.443 to 1.230]), or recurrent ischemic events (eg, recurrent event≈arterial PI: OR, 1.196 [95% CI, 0.454–3.168]; Table 5). However, patients who had a recurrent ischemic event had lower baseline venous sinus PI (OR, 0.125 [95% CI, 0.015–0.897]), lower CSF stroke volume (OR, 0.300 [95% CI, 0.110–0.794]), and lower peak CSF flow associated with worse 1-year mRS (OR, 0.757 [95% CI, 0.605–0.941]).

## Discussion

We investigated potential cross-sectional and longitudinal relationships between intracranial arterial, venous, and CSF flow and pulsatility measures, baseline and 1-year SVD features, and clinical outcomes. We found that patients with higher arterial stiffness had larger baseline WMH and PVS volumes but only limited associations between pulsatility measures and 1-year imaging or clinical outcomes.

### Intracranial Vascular Stiffness Measures

#### Cross-Sectional

Patients with larger WMH and PVS volumes had higher arterial pulsatility across several stiffness measures (PI, RI, and, for WMH volume, FVP), reflecting recent cross-sectional SVD studies.^5,8^ PIs of cerebral arteries have previously been shown to have a positive association with carotid-femoral PWV in healthy older adults,^39^ which itself is positively associated with cross-sectional and longitudinal SVD severities.^40^ Arterial pulsatility and WMH/PVS burden associations may partially reflect a coassociation with age but remain after age adjustment.^41^ Previously, higher arterial FVP, but not PI, was reported to be strongly associated with higher WMH volume.^5^ However, 4D PC-MRI sacrifices spatiotemporal resolution for acquisition volume^42^ versus 2D PC-MRI; hence, a greater partial volume effect in 4D PC-MRI may change the waveform, potentially explaining the disparity.^43^

Unlike previous findings,^8,30^ we saw little association between venous sinus PI and SVD features. Hypotheses put forward include systolic arterial expansion producing pressure waves within the CSF spaces that are transmitted to the venous sinuses and increased capillary pulsatility (due to deficient dampening in the arteries) transmitted to the veins through blood flow. Increased venous sinus pulsatility may result from poor dampening upstream of the capillaries.

Intracranial PTT showed little relation to WMH volume, reflecting previous findings.^8^ We saw longer arterial-CSF PTTs in patients with larger CSO PVS volumes, suggesting a possible link between altered pulse propagation through the brain and enlarged PVS. Intracranial PTTs encompass several vascular beds and possess lower temporal resolution than PWV, which may measure propagation more accurately.^44^ When assessing PTT, simultaneous measurement of vessels is preferable (as this allows for a true, synchronized snapshot of waveform propagation, avoiding errors introduced by temporal changes), but, due to flow velocity differences that affect the selection of velocity encoding, this was not technically viable using available PC-MRI methods. Our exclusion from PTT analysis of patients with notable heart rate disparities between sequences and normalizing cardiac cycles to 1 second should mitigate this limitation.

Stronger associations between arterial pulsatility and BG versus CSO PVS volume are consistent with previous findings,^8,45^ perhaps as BG PVS more strongly relates to higher BP/pulse pressure^45^ or due to their proximity to larger blood vessels.

#### Longitudinal

Neither baseline arterial nor venous pulsatility is associated with 1-year SVD score, WMH, or PVS volume, despite clear SVD burden changes, including WMH volume increases and decreases. Previous studies that found higher PWV with WMH progression^46,47^ measured PWV in large systemic not intracranial arteries as measured here.

Intracranial vessel pulsatility showed no clear associations with 1-year cognitive function or dependency. Greater 1-year decreases in cognitive function after acute stroke/transient ischemic attack were previously associated with increased middle cerebral artery but not ICA pulsatility (4D PC-MRI; N=89).^5^ We did, surprisingly, find that patients with lower baseline venous sinus and CSF pulsatility had higher 1-year odds of a recurrent ischemic event. Lower venous and CSF pulsatility could relate to impaired brain waste clearance, which could be associated with a worse disease burden and, therefore, a greater risk of stroke. The role of venous and CSF pulsatility in brain health requires further investigation.

While several cross-sectional studies^6^ found clear associations between higher pulsatility and worse SVD, mirroring our cross-sectional results, large longitudinal studies are scarce. Our findings are consistent with a recent smaller study in N=122 healthy older subjects, suggesting that higher pulsatility does not predict 5-year WMH and PVS growth.^11^ Indeed, a converse relationship was found although we cannot substantiate this due to lacking longitudinal pulsatility measurements.

### Cerebral Blood Flow

We found sparse evidence of associations between CBF and SVD features though lower baseline CBF is weakly associated with larger 1-year PVS volume. While associations between CBF and longitudinal PVS volume change have not been explored previously to our knowledge, previous large-scale systematic reviews^48,49^ reported small studies showing cross-sectional associations between CBF and WMHs that attenuated in larger studies.^49^ The few longitudinal studies mostly found that high WMH volumes predicted low CBF longitudinally,^9,49^ suggesting that low CBF is a consequence, not a cause, of SVD-related damage, generally consistent with our findings.

Estimating global CBF from the combined internal carotid and vertebral arterial inputs has limitations because it cannot account for contributions from other, small arteries. However, it is a reasonable proxy measure of global CBF that enables comparisons across the cohort when applied in a consistent manner, as done here. Other methods to measure CBF also have limitations; for example, for ASL, labeling efficiency can vary in patients with vascular changes, and many methods exclude the posterior fossa, thereby underestimating total CBF. Several studies have previously assessed regional CBF associations with vascular pathology,^49^ with results indicating no clear cross-sectional relationship between WMH volume and CBF in normal-appearing white matter or gray matter, while lower CBF in the periventricular WMH penumbra was associated with WMH growth in healthy older participants. Therefore, it was not our intention here to measure regional CBF, which would be difficult to relate to arterial or venous pulsatility measures but to provide a global measure of CBF as a background setting for the pulsatility measures. In addition, normalizing each participant’s CBF measurement to their brain volume accounted for differences in brain size and atrophy, enabling more accurate comparisons.

### CSF Measures

We found some associations between worse mRS, lower CSF stroke volume, and peak flow (potentially due to a link between impaired brain waste clearance affecting cognition), but CSF flow and pulsatility generally showed little association with SVD features (except as below). Enlarged PVSs suggest sluggish interstitial fluid movement, but exact mechanisms remain unclear. The lack of associations is consistent with some previous findings^8,17^ but not others.^15,16^ One previous study^16^ used amplitude transfer functions to describe CSF pulse waves, which could explain the difference. We previously found that CSF stroke volume and PVS score were weakly associated but did not assess PVS volume.^30^

We found a 2.23-mL/s net foramen magnum CSF flow rate cranially, perhaps due to measurement errors, pathology, or net CSF movement to drainage pathways primarily exiting from the skull (cribriform plate, meningeal, perivascular, and perineural).^50^ Interestingly, we found a small trend between higher peak CSF flow and larger 1-year WMH volume, while lower CSF stroke volume and peak flow were associated with higher 1-year mRS score, suggesting that CSF dynamics may be associated with SVD-related dependency. Animal models exhibit a relationship between CSF, brain interstitial fluid flow, and waste clearance,^14^ but the exact relationship in humans and possible links with SVD remain undetermined.

### Strengths and Weaknesses

Strengths of this study include the large (>200) sample, highly phenotyped clinically, for baseline and 1-year SVD features using well-established methods, assessing PVS and WMH, contemporaneous arterial, venous and CSF flow analyses, multiple vessel stiffness/pulsatility measures, clinical outcomes, and a relevant control group that accounts for medication. Currently, this is the largest such longitudinal study. We did not include a healthy control group because it would not account for medication effects, or comorbidities, and would add little^2^; instead, we recruited patients with nonlacunar strokes as controls for lacunar (SVD) stroke and a broad range of disease burdens. Weaknesses include the lack of follow-up PC-MRI and the limited PC-MRI spatial and temporal resolutions, meaning that only a limited number of vessels could be assessed, in particular smaller, penetrating vessels were not examined. Longer follow-up duration may be needed to detect some SVD-related outcomes and should include regional CBF measures. In the future, we may explore the relationship between pulsatility and BP variation and analyze structural images over longer follow-up periods.

### Conclusions

Despite clear cross-sectional associations between pulsatility and SVD, neither baseline intracranial arterial, venous, or CSF pulsatility nor CBF had major effects on 1-year clinical or imaging outcomes. As a recent smaller study with longer follow-up suggested, pulsatility and stiffness may result from, rather than cause, SVD damage. Imaging in early SVD stages may be needed to identify predictive pulsatility measures. Further research should assess the order of these changes, whether pulsatility differs between patients with SVD progression versus regression, and how we may intervene in SVD progression.

## Article Information

### Acknowledgments

The authors thank Agnieszka Czechon, Rachel Locherty, and the Edinburgh Imaging radiographers.

### Sources of Funding

This work was supported by Medical Research Scotland Studentship (to Dr Morgan; grant PhD-1165–2017) partnered with Siemens Healthineers; Siemens; UK Dementia Research Institute that receives its funding from DRI Ltd, primarily funded by the UK Medical Research Council and Alzheimer’s Society; Fondation Leducq Network for the Study of Perivascular Spaces in Small Vessel Disease (16 CVD 05); Stroke Association Post-Doctoral Fellowships (to Drs Stringer and Wiseman; grants SAPDF 23/100007 and 18/100026), Small Vessel Disease-Spotlight on Symptoms (grant SAPG 19\1000680), and Garfield Weston Foundation Senior Clinical Lectureship (to Dr Doubal; grant TSALECT 2015/04); The Mrs Gladys Row Fogo Charitable Trust Center for Research into Aging and the Brain; NHS Research Scotland (to Dr Doubal); the British Heart Foundation Edinburgh center for Research Excellence (grant RE/18/5/34216); the NHS Lothian Research and Development Office (to Dr Thrippleton); the European Union Horizon 2020, PHC-03–15, project No666881, SVDs@Target (to Dr Stringer); the Chief Scientist Office of Scotland Clinical Academic Fellowship (to Dr Clancy; grant CAF/18/08); the Stroke Association Princess Margaret Research Development Fellowship (to Dr Clancy; grant 2018), and CONACYT (Consejo Nacional de Ciencia y Tecnología; National Council of Science and Technology), the Rowling Clinic and Row Fogo Charitable Trust as above (to Dr Arteaga Reyes); Medical Research Council (National Productivity Fund MR/R502327/1; to Dr Sleight); Wellcome Trust Translational Neuroscience Ph.D. Programme (grant 224912/ Z/21/Z; to Dr Jamie Garcia); the Alzheimer’s Society (ref 486 [grant AS-CP-18b-001]); the University of Edinburgh College of Medicine and Veterinary Medicine (to Dr Jochems); and the Scottish Funding Council through the Scottish Imaging Network: A Platform for Scientific Excellence Collaboration. The 3T scanner is funded by the Wellcome Trust (grant 104916/Z/14/Z), the Dunhill Trust (grant R380R/1114), the Edinburgh and Lothians Health Foundation (grant 2012/17), the Muir Maxwell Research Fund, and The University of Edinburgh.

### Disclosures

Siemens Healthineers provided partial PhD studentship support (to Dr Morgan) but had no role in the study. Prof Wardlaw reports academic grants that funded the research as listed in the Sources of Funding, which had no role in the study design, conduct, analysis, or interpretation, including the Wellcome Trust, the Medical Research Council, the Stroke Association, the Biotechnology and Biological Sciences Research Council, the Weston Brain Institute, the Alzheimer’s Society, Fondation Leducq, the Horizon 2020 Framework Programme, Chief Scientist Office, the Dunhill Medical Trust, the Mrs Gladys Row Fogo Charitable Trust, and the British Heart Foundation. The other authors report no conflicts.

### Supplemental Material

Supplemental Methods

Table S1
